# Molecular Characteristics and Efficacy of *16D10* siRNAs in Inhibiting Root-Knot Nematode Infection in Transgenic Grape Hairy Roots

**DOI:** 10.1371/journal.pone.0069463

**Published:** 2013-07-16

**Authors:** Yingzhen Yang, Yingyos Jittayasothorn, Demosthenis Chronis, Xiaohong Wang, Peter Cousins, Gan-Yuan Zhong

**Affiliations:** 1 United States Department of Agriculture-Agricultural Research Service, Grape Genetics Research Unit, Geneva, New York, United States of America; 2 Laboratory of Immunology, National Eye Institute, National Institutes of Health, Bethesda, Maryland, United States of America; 3 United States Department of Agriculture-Agricultural Research Service, Robert W. Holley Center for Agriculture and Health, Ithaca, New York, United States of America; 4 E & J Gallo Winery, Modesto, California, United States of America; University of Tennessee, United States of America

## Abstract

Root-knot nematodes (RKNs) infect many annual and perennial crops and are the most devastating soil-born pests in vineyards. To develop a biotech-based solution for controlling RKNs in grapes, we evaluated the efficacy of plant-derived RNA interference (RNAi) silencing of a conserved RKN effector gene, *16D10*, for nematode resistance in transgenic grape hairy roots. Two hairpin-based silencing constructs, containing a stem sequence of 42 bp (*pART27-42*) or 271 bp (*pART27-271*) of the *16D10* gene, were transformed into grape hairy roots and compared for their small interfering RNA (siRNA) production and efficacy on suppression of nematode infection. Transgenic hairy root lines carrying either of the two RNAi constructs showed less susceptibility to nematode infection compared with control. Small RNA libraries from four *pART27-42* and two *pART27-271* hairy root lines were sequenced using an Illumina sequencing technology. The *pART27-42* lines produced hundred times more *16D10*-specific siRNAs than the *pART27-271* lines. On average the *16D10* siRNA population had higher GC content than the *16D10* stem sequences in the RNAi constructs, supporting previous observation that plant dicer-like enzymes prefer GC-rich sequences as substrates for siRNA production. The stems of the *16D10* RNAi constructs were not equally processed into siRNAs. Several hot spots for siRNA production were found in similar positions of the hairpin stems in *pART27-42* and *pART27-271*. Interestingly, stem sequences at the loop terminus produced more siRNAs than those at the stem base. Furthermore, the relative abundance of guide and passenger single-stranded RNAs from putative siRNA duplexes was largely correlated with their 5′ end thermodynamic strength. This study demonstrated the feasibility of using a plant-derived RNAi approach for generation of novel nematode resistance in grapes and revealed several interesting molecular characteristics of transgene siRNAs important for optimizing plant RNAi constructs.

## Introduction

Root-knot nematodes (RKNs) (*Meloidogyne* sp.) infect a wide range of plant species, including grapes (*Vitis vinifera* L.) and many other important crops. RKNs are widely distributed in vineyard soils in many countries [1]. The impact of RKNs on grape growth and production are especially severe for grapes grown on warm sandy soils [2]. For example, in Australia, almost all vineyards on sandy soils were infected with RKNs. RKNs may occur in more than half of the California vineyard acreage which accounts for 90% of US grape production [1]. Nematode management through fumigation, particularly by using methyl bromide, can be useful in mitigating infestation. However, the use of chemicals is not only costly but also environmentally harmful. The most cost-effective solution to control RKNs in vineyards is to use nematode resistant rootstocks. Resistant rootstock varieties, such as Freedom, Harmony, Dog Ridge, and Ramsey, have been successfully deployed in vineyards to suppress nematode infestation. Nevertheless, these decades-old rootstock varieties, while still effective, have been becoming vulnerable to infection by emerging aggressive RKN populations [1]–[4]. Breeding for nematode-resistant rootstocks is a long and laborious process, which may take more than one decade to obtain commercially acceptable resistant rootstocks. Furthermore, the occurrence of multiple RKN species and the constant emergence of new virulent populations in vineyards make the conventional breeding for nematode resistant rootstocks even more challenging. Molecular marker technologies can accelerate the breeding process through identification and use of the markers closely linked to the genes or QTLs controlling root-knot nematode resistance, as demonstrated for resistance to dagger nematodes [5], [6].

Successful nematode parasitism depends on its secreted effectors that function to overcome plant defense and to induce and maintain feeding cells [7]. Recently, significant research progress has been made in using an RNA interference (RNAi) technology to silence the expression of these nematode effector genes, thus inhibiting or compromising nematode parasitism. The basic idea is to introduce into host plants an expression cassette producing double stranded RNAs (dsRNAs) targeting one or multiple nematode genes that are critical for nematode infection. When nematodes feed on the roots of the engineered hosts they then ingest dsRNAs and/or dsRNA-derived small interfering RNAs (siRNAs) that subsequently result in the suppression of target gene expression, thereby impairing successful nematode parasitism [8]. Such strategy has been successfully demonstrated in transgenic *Arabidopsis* [9], [10], tobacco [11] and soybean [12] for controlling RKNs; and in *Arabidopsis* [13] and soybean [14]–[16] for controlling cyst nematodes. However, in spite of these successes, there are many remaining challenges in optimizing various components of RNAi constructs, such as effector gene selection and dsRNA stem length in the hairpin structure, for enhancing RNAi efficacy [8].

In animal RNAi research, short hairpin RNAs (shRNAs) with stem length of 19 to 30 bp were often used due to the possible interferon response induced by long dsRNAs [17]. Studies in mammalian cells suggested that shRNAs with short stems (19 or 21 bp) were most effective in silencing target genes [18], [19] and siRNA production was much reduced when the stem length exceeded beyond 33 bp [20]. Another study showed that, when shRNAs with stem length varying from 42 to 93 bp were compared, shRNAs with stem length longer than 66 bp were less efficient in producing siRNAs than the ones with shorter stems [21]. In plants, however, such direct comparisons of long and short stems were rarely reported and, in general, constructs for RNAi research in plants contain longer hairpin stem than that in animals. RNAi constructs with stem length varying from 80 bp to more than 1000 bp have been reported to generate RNAi silencing signals for suppressing a target gene in plant RNAi research [17].

siRNA generation from dsRNAs/hairpins involves complex molecular processes. Dicer-like enzymes process dsRNAs into 19–26 bp siRNA duplexes with a typical 3′ overhang of two nucleotides [22]–[25]. An siRNA duplex is composed of one guide single-stranded RNA (ssRNA) and one passenger ssRNA. Guide ssRNAs are complementary to mRNA sequences of target genes and act as guides in RNA-induced silencing complexes (RISC) for sequence-specific mRNA cleavage and translational repression. Passenger ssRNAs share the same orientation as the mRNA and are not responsible for target-specific gene regulation [24], [25]. Extensive studies have been conducted in mammalian cells to evaluate various molecular properties of siRNA duplexes for their silencing efficacy. Important properties include asymmetric 5′ end thermodynamic strength, modest GC content, sense strand base preferences at certain positions, and lack of secondary structure in the target mRNA for easy target access [26]–[29]. However, prediction programs developed on the basis of these parameters do not necessarily pick up the most potent siRNA sequences [30], indicating the presence of some other important factors yet to be discovered. One obvious but important factor is that not all genes or fragments in the same gene are equally targeted by RNAi [27]. Because of the potential opportunities of RNAi application in gene therapy, significant effort has been directed toward optimizing siRNA duplex sequences for achieving high potency, low toxicity and low off-target effect in medical research [30], [31]. In contrast, plant RNAi research in these areas is much behind and we know very little about various molecular properties of siRNAs generated from an introduced RNAi construct. Does hairpin stem length matter in terms of siRNA production? How well is dsRNA expression level correlated with siRNA abundance? What kinds of siRNAs are produced from a hairpin construct? Are guide and passenger ssRNAs equally produced? Answering these questions will help better understand various properties of plant RNAi and design RNAi constructs with high silencing efficacies. Some of these questions can be addressed by carefully examining relevant small RNA profiles as was demonstrated in this study.

In this study we evaluated the feasibility of generating RKN resistance via a hairpin-based RNAi technology in transgenic grape hairy roots. A conserved RKN effector gene, *16D10*, was selected as the RNAi target. The *16D10* gene codes for a secretory peptide of 13 amino acids in RKN subventral esophageal gland cells [9], [32]. It plays an important role in establishing feeding sites for the endoparasitic RKNs and knockdown of *16D10* gene expression in RKNs by RNAi offered a broad spectrum of resistance against all the major RKN species in transgenic *Arabidopsis* plants [9]. Our study extended the *16D10* findings in the model species *Arabidopsis* to grape, an economically important fruit crop, and advanced our knowledge in using a genetic engineering approach for controlling RKNs in vineyards. We evaluated two hairpin constructs, one with a 42 bp stem and the other with a 271 bp stem of the *16D10* gene. The small RNA profiles derived from both short and long hairpin constructs were examined using the next-generation sequencing technology. The results obtained in this study provided important insights into the processes of siRNA generation, selection and preservation of guide and passenger ssRNAs and optimization of RNAi construct design in plant RNAi research and application.

## Materials and Methods

### Generation of *16D10* dsRNA Constructs

The *16D10* gene was provided by Dr. Richard Hussey of University of Georgia. Two binary *16D10* dsRNA constructs were generated following the same method as previously described by Huang et al. [9]. These two constructs differed in their hairpin stem length. One had a short stem consisting of a 42 bp core-coding sequence covering 13 amino acids of the mature 16D10 peptide and a stop codon. The other had a long stem of 271 bp containing a 42 bp 5′ untranslated region (UTR), a 132 bp coding region, and a 97 bp 3′UTR. These sequences were separately cloned into a *pHANNIBAL* vector as reverse tandem repeats [33]. The resulting *35S::dsRNA* cassette containing short- or long- stem from the *pHANNIBAL* vector was then cloned into a *pART27* binary vector, which contains an *in planta* kanamycin selection marker, to generate the *16D10* dsRNA constructs of *pART27-42* (short-stem) or *pART27-271* (long-stem). An empty *pART27* binary vector was used as control.

### Generation of Transgenic Grape Hairy Roots

*V. vinifera* cv. Chardonnay, a well-known wine grape susceptible to RKN infection, was used as transformation material in this study. Transgenic hairy roots were generated following the stem-cut surface protocol as described by Jittayasothorn et al. [34]. *Agrobacterium rhizogenes* strain A4 was transformed with *pART27*, *pART27-42* or *pART27-271* construct. The transformed *Agrobacterium* clones were confirmed by colony polymerase chain reaction (PCR) for the presence of a target construct. Young shoots of *in vitro* plants were cut at the stem ends and inoculated with *Agrobacterium* culture. After two weeks of co-cultivation, the shoots were washed and transferred to bacteria removing medium. Within three to five weeks, vigorously growing whitish hairy roots appeared from calli. The hairy roots were then isolated and transferred to hairy root culture medium supplemented with 20 mg/L kanamycin to select positive transgenic individuals. Each survival hairy root was established as an independent line and was tracked by its original identity. Genomic PCRs were used to confirm the presence of the RNAi cassette in the transgenic hairy root lines. These hairy root lines were cultured for four to six weeks, then primary and secondary root tips (1–1.5 cm long) were transferred to fresh plates supplemented with kanamycin to multiply the hairy roots. Hairy root lines that succeeded with multiple subcultures with enough secondary hairy roots were used in the evaluation of RKN resistance.

### Evaluation of Transgenic Hairy Roots for RKN Resistance

Young and healthy transgenic hairy root tips (1–1.5 cm long) were harvested for individual lines and transferred to nematode testing plates (hairy root culture medium with 1% Agar and 100 mg/L cefotaxime). Plates were placed semi-vertically (∼70–80 degree) and incubated at room temperature (20–22°C) in the dark. Four days after transferring hairy root tips to nematode testing medium, *Meloidogyne incognita* race 3 eggs were collected from tomato plants cultured hydroponically [35]. The nematode eggs were sterilized with 0.02% sodium azide (Sigma-Aldrich) for 20 minutes. The sterilized eggs were rinsed with distilled water and transferred to hatching solution (0.1 mg/ml nystatin (Sigma-Aldrich) and 1.5 mg/ml gentamicin (Sigma-Aldrich)) in a hatching pan. The eggs were hatched at room temperature for three days. The hatching mixture containing infectious J2 nematodes was poured into a 50 ml Falcon tube and settled for one hour. Infectious J2 nematodes at the bottom of the tube were transferred to a 1.5 ml low-adhesion tube and sterilized in 1 ml sterilization solution (0.004% mercuric chloride (Acros) and 0.004% sodium azide) for 10 minutes. The sterilized J2 nematodes were collected by centrifugation and washed five times with sterile water. The J2 nematodes were counted under a microscope and re-suspended in sterilized 0.1% agarose to a final concentration of 20–30 J2 nematodes per 10 µl solution. Each hairy root was inoculated with 20–30 sterilized *M. incognita* J2 nematodes (10 µl) at a place 0.5 cm from the root tip end. Pictures were taken at 0, 2, 3, 4 and 5 weeks post inoculation to monitor gall formation and hairy root growth. Nematode eggs were extracted from hairy roots five weeks after nematode inoculation using a bleaching/blending method [36]. To visualize nematodes in the transgenic hairy roots, the infected roots were stained using the sodium hypochlorite-acid fuchsin method previously reported [37].

Transgenic hairy roots were evaluated against RKN infection in three independent experiments. In addition to general observation, data for fresh weight of infected hairy roots and number of nematode eggs were collected. In the first experiment, data were collected on a plate basis with hairy roots on the same plates being pooled for RKN egg extraction. Each plate contained 3–6 hairy roots. Means of fresh hairy root weight (mg), number of eggs per hairy root, and number of eggs per gram root were calculated accordingly. In the second and third experiments, data were collected for individual hairy roots. The number of hairy roots evaluated for individual transgenic lines varied, depending on the availability of hairy roots for individual lines. For the same reason, some lines were evaluated in only two experiments instead of three. When a hairy root line, including the control, had more than 4 hairy roots available for evaluation in the second or third experiment, only the top 4 with the severest nematode infection (largest numbers of eggs per hairy root) were included in data analysis. By doing so, we hope to reduce the potential confounding effect of false positive data points on intra-line variation due to inoculation escape and/or other factors which might compromise the effectiveness of nematode infection. The three experiments were treated as independent replicates in data analysis, with each hairy root line having 1–4 observations in each experiment. To reduce skewness of data distribution, the raw data were transformed using log_10_ and then analyzed using the General Linear Model program of SAS (version 9.2) in which replicate, construct and line effects were respectively estimated and tested. The significant difference between the means of individual hairy root line and control was determined on the basis of Dunnett’s T-test at 0.01 level.

### Small RNA Library Preparation and Sequencing

Five weeks after nematode infection, secondary hairy root tips were cut and transferred to fresh hairy root culture medium supplemented with 100 mg/L cefotaxime and 20 mg/L kanamycin in order to multiply hairy roots for RNA extraction. Root tips approximately 1 cm long from three-week old culture were collected and low-molecular-weight RNAs were extracted using a cetyltrimethylammonium bromide-based method [38]. The protocol for constructing small RNA libraries was provided by Dr. Silin Zhong at the Boyce Thompson Institute, Cornell University. Small RNAs in the range of 15–30 nts were purified using 15% Tris/Borate/EDTA Urea polyacrylamide gel. 3′ universal miRNA cloning linkers (5′ rAppCTGTAGGCACCATCAAT-NH_2_ 3′) were ligated to the small RNAs using T4 RNA ligase 2 truncated (New England Biolab) overnight at 18°C. The ligated small RNAs with the 3′ linker were purified using 10% Tris/Borate/EDTA Urea polyacrylamide gel. The purified small RNAs with 3′ linker were then ligated with the 5′ linkers (5′GUUCAGAGUUCUACAGUCCGACGAUC 3′) using T4 RNA ligase 1 (New England Biolab) overnight at 18°C. Reverse transcription (RT) was carried out with Superscript III (Invitrogen) using an RT primer (5′ GATTGATGGTGCCTACA 3′). The RT products were purified by ethanol precipitation and used as templates for PCR amplification of the small RNA libraries. Universal primer (AATGATACGGCGACCACCGAGATCTACACGACAGGTTCAGAGTTCTACAGTCCGA) and barcoded primer (CAAGCAGAAGACGGCATACGAGAT*NNNNNN*gattgatggtgcctacag) (“*NNNNNN*” stands for the barcode sequence) were used to amplify each individual small RNA library with Phusion DNA polymerase (New England Biolab). The small RNA libraries were purified using a 2% agarose gel (with 0.01% SYBR safe (Invitrogen)) in LB buffer (Faster Better Media). The library DNA fragments (around 125–150 bp) were purified using a Qiagen MinElute gel extraction kit. Each individual small RNA library was quantified using an Invitrogen Qubit fluorometer with the dsDNA high-sensitivity assay kit (Invitrogen). Equal amount of small RNA libraries for different hairy root lines were mixed together to form a pooled small RNA library. The pooled small RNA library was sequenced with miRNA primer using the Illumina sequencing platform (HiSeq2000, short read) provided by the Cornell University Life Sciences Core Laboratories Center.

### Small RNA Analysis

CLC Genomic Workbench software (CLC bio, Cambridge, MA) was used to process the sequence data generated by Illumina HiSeq. Briefly, the fastq file was imported into CLC genomic workbench. In a raw read such as “**TAGTGGGCCAAATCCTGGAGG**ctgtaggcaccatcaatc*ACATCG*ATCTC”, the sequence in bold is the small RNA sequence, the sequence in lower case is derived from the small RNA 3′ linker/the RT primer, and the sequence in italic is the barcode sequence embedded in the PCR barcode primer. Small RNA 3′ linker/RT primer-derived sequence “gattgatggtgcctacag” was used for custom adaptor trimming. About 84% of the total reads (187,608,102) was retained after the adaptor trimming. About 0.14% of the total reads were mapped to the *16D10* gene (225,344). The mapped reads were extracted and counted and then exported to an Excel spreadsheet for manually assigning the reads to individual transgenic hairy root lines according to their line-specific barcodes used for constructing small RNA libraries. Reads with perfect adaptor and barcode sequences (91.5%, 206,465 reads) were analyzed further. The read count, origin, and orientation (guide or passenger ssRNA) of each small RNA species were summarized.

## Results

### *16D10* dsRNAs Inhibited RKN Infection in tTransgenic Grape Hairy Roots

**Figure 1:**
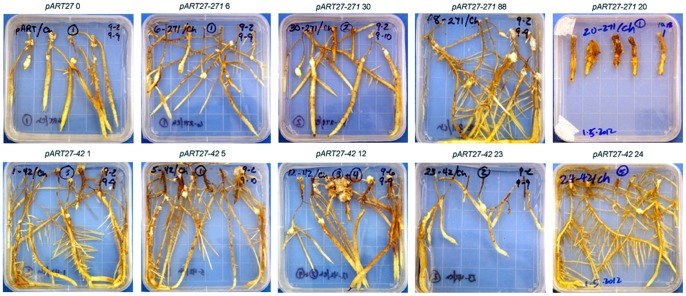
Representative transgenic grape hairy root lines used in this study. Individual hairy root lines carrying a *16D10* RNAi construct, *pART27-42* or *pART27-271*, were cultured and inoculated with J2 RKNs to evaluate their resistance against RKNs. *pART27 0* was a control line which was transformed with an empty binary vector *pART27*. Note that *pART27-271* line 20 and *pART27-42* line 24 showed contrasting variation in their root morphology and proliferation. The pictures were taken three weeks (*pART27-271* line 20 and *pART27-42* line 24) or five weeks (the rest) after nematode inoculation.

More than 20 independent transgenic hairy root lines were generated for both *pART27-42* and *pART27-271* constructs. There was a wide range of variation in morphology and growth vigor among these hairy root lines. Some lines were thick (up to 5 mm in diameter) while some lines were very thin (less than 1 mm in diameter); and some lines produced many secondary hairy roots while some lines rarely produced any, which eventually led to the loss of the lines (Figure 1). We selected uniform hairy roots for RKN infection when possible.

Several criteria have been used to assess severity of RKN infection in RKN research, including gall number and size, number of nematodes, developmental stages of the nematodes, and number of eggs [9]–[12], [39]. We chose to use the numbers of RKN eggs per hairy root and per gram root as the indicators of nematode resistance, as the number of nematode eggs directly measures the success of nematode reproduction.

**Figure 2:**
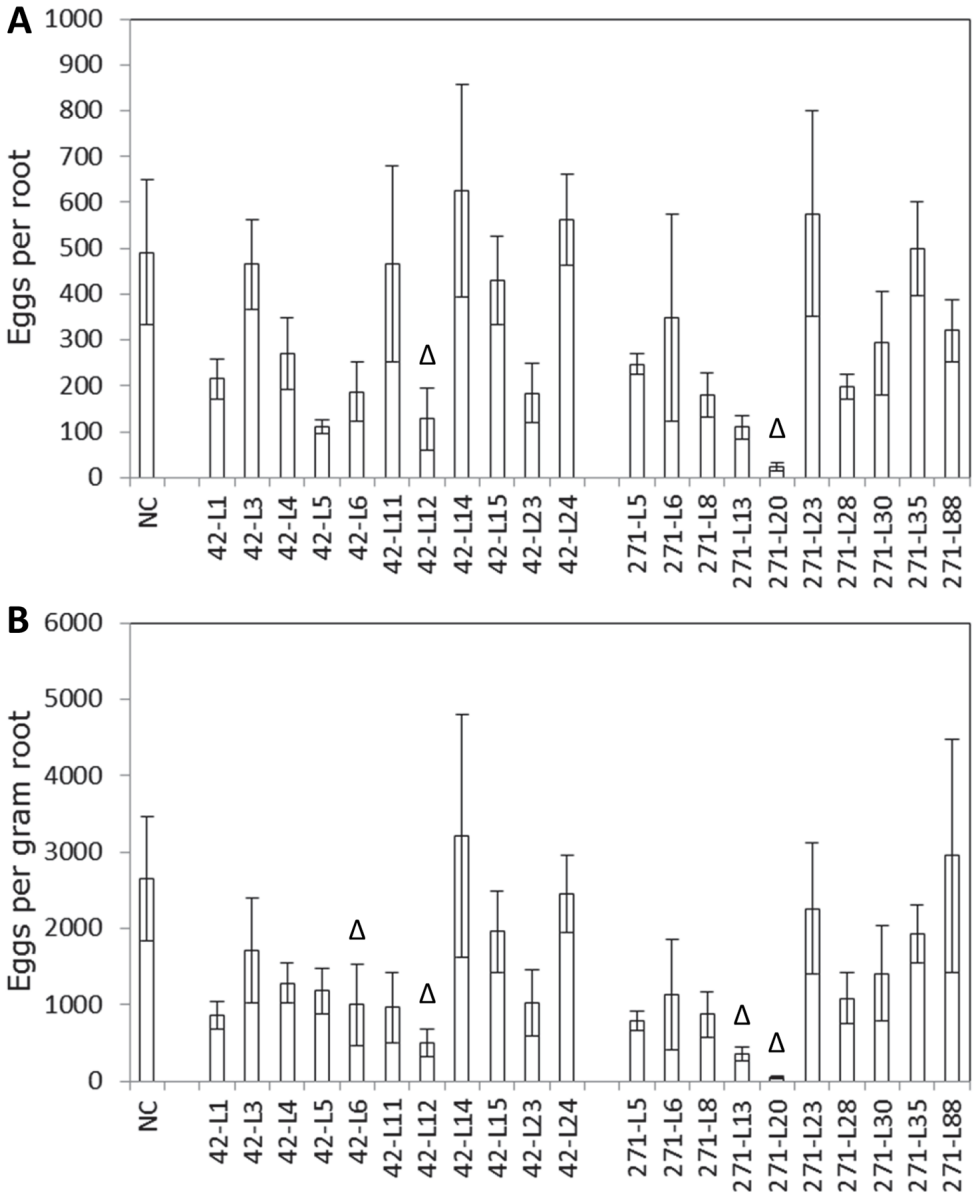
Reproduction of root-knot nematodes on *16D10* transgenic hairy root lines. (A) Eggs per hairy root. (B) Eggs per gram hairy root. NC is the negative control (*pART27* 0). 42-L1 and 271-L5 represent the abbreviations of *pART27-42* line 1 and *pART27-271* line 5, respectively. Bars represent the means±SEs observed from individual hairy root lines. Bars (hairy root lines) with “Δ” were significantly different from the negative control at P<0.01, on the basis of log_10_ transformed data. Data for this figure were provided in Table S1.

Eleven independent hairy root lines of *pART27-42* and ten lines of *pART27-271* constructs were selected for evaluation of RKN resistance in this study (Figure 2 and Table S1). The means and standard errors of the raw and log_10_ transformed data of the fresh weight, egg number per hairy root, and egg number per gram root for these lines were summarized (Table S1). There was a large range of variation in the fresh weight (203.6 to 550.0 mg), number of eggs per hairy root (24.0 to 626.8), and number of eggs per gram root (54.8 to 3209.0). A simple correlation analysis across all lines indicated that there was no significant correlation between the fresh weight of hairy root and the number of eggs per hairy root. However, fresh weight had a significantly negative correlation with the number of eggs per gram root (- 0.559, P<0.01). On the other hand, the numbers of eggs per hairy root and per gram root were highly correlated (0.849, P<0.01). Among 11 *pART27-42* lines evaluated, lines 6 and 12 showed significantly better resistance to RKNs than the control (Figure 2 and Table S1). The numbers of eggs per hairy root and per gram root for the control were 491.4 and 2655.2, respectively. Compared with the control, line 6 had significantly lower number of eggs per gram root (1001.6, P<0.01). Similar result was observed for line 12, which had lower numbers of eggs per hairy root (128.1, P<0.01) and per gram root (499.8, P<0.01) than the control. Lines 5 and 23 also had lower numbers of eggs per hairy root than the control, although statistically not significant at P<0.01. Among the 10 *pART27-271* hairy root lines, lines 13 and 20 had significantly better resistance to RKNs than the control. The number of eggs per gram root for line 13 was 357.2, which was significantly lower than the control at P<0.01. Line 20 produced only 24.0 eggs per hairy root and 54.8 eggs per gram root. Compared with the control, both these two numbers were significantly lower than that of the control at P<0.01 level (Figure 2 and Table S1). It is interesting to note that line 20 exhibited the strongest RKN resistance as indicated by the smallest number of eggs produced per hairy root and per gram root. However, its primary hairy roots were very thick and produced very few secondary hairy roots (Figure 1). The precise nature of this line showing such a high level of RKN resistance, compared with the other *pART27-271* lines, was unknown, but the unusual thick hairy roots and low hairy root proliferation rate suggested that this was probably an exceptional case. Unfortunately we could not evaluate this line further due to the difficulty in obtaining sufficient secondary roots for additional nematode tests and RNA extraction.

### The *pART27-42* Lines Produced more *16D10*-specific siRNAs than the *pART27-271* Lines

To verify that *16D10*-specific siRNAs were produced in the transgenic hairy root lines, small RNA libraries were prepared for four *pART27-42* and two *pART27-271* lines (Table 1). The small RNA libraries were barcoded and pooled for Illumina miRNA sequencing. About 180 million reads were generated. A total of 225,344 reads were mapped to the *16D10* gene with an average length of 20.8 nucleotides (nts). The length of siRNAs ranged from 15 to 25 nts. The 21-nt siRNAs were most abundant (71.8%), followed by 20-nt siRNAs (6.6%) and 24-nt siRNAs (6.2%). The first 100 most abundant small RNA species (designated as R1 to R100) covered 92.7% of the total reads (Tables 2 and S2). The top ten small RNAs were all 21-nt long and counted for 66% of the total *16D10*-specific small RNAs (Table 2).

**Table 1:** Numbers of *16D10* small RNA reads observed from four *pART27-42* and two *pART27-271* transgenic hairy root lines.

Hairy root line	*pART27-42* Line 1	*pART27-42* Line 5	*pART27-42* Line 12	*pART27-42* Line 23	*pART27-271* Line 30	*pART27-271* Line 88
Guide ssRNA	17765	21017	22221	16629	27	19
Passenger ssRNA	34791	19105	47243	27538	65	43
Guide/passenger	0.51	1.1	0.47	0.6	0.42	0.44

**Table 2:** Sequences, GC content and reads for the top 50 small RNAs from four *pART27-42* hairy root lines.

SmallRNAID	small RNA sequence	Sequencelength	GC%	No. of GuidessRNA	No. of PassengerssRNA	Guide/passengerRatio
16D10	GGCAAAAAGCCUAGUGGGCCAAAUCCUGGAGGAAAUAAUUGA	42	45.2			
R1	UAGUGGGCCAAAUCCUGGAGG	21	57.1	643	78930	0.01
R2	CCUAGUGGGCCAAAUCCUGGA	21	57.1	9745	800	12.18
R3	CUAGUGGGCCAAAUCCUGGAG	21	57.1	1393	6340	0.22
R4	AGCCUAGUGGGCCAAAUCCUG	21	57.1	6704	703	9.54
R5	AAAGCCUAGUGGGCCAAAUCC	21	52.4	6414	6	1069
R6	AAUCCUGGAGGAAAUAAUUGA	21	33.3	1649	4657	0.35
R7	GUGGGCCAAAUCCUGGAGGAA	21	57.1	5603	90	62.26
R8	UGGGCCAAAUCCUGGAGGAAA	21	52.4	5100	216	23.61
R9	AAGCCUAGUGGGCCAAAUCCU	21	52.4	3981	70	56.87
R10	GGCCAAAUCCUGGAGGAAAUA	21	47.6	3375	118	28.6
R11	AGUGGGCCAAAUCCUGGAGG	20	60	75	3197	0.02
R12	AAAUCCUGGAGGAAAUAAUUG	21	33.3	136	2439	0.06
R13	GCCAAAUCCUGGAGGAAAUAA	21	42.9	2231	149	14.97
R14	GGGCCAAAUCCUGGAGGAAAUAAU	24	45.8	2308	21	109.9
R15	UGGGCCAAAUCCUGGAGGAAAUAA	24	45.8	1978	16	123.63
R16	AAUCCUGGAGGAAAUAAUUG	20	35	99	1693	0.06
R17	AAAGCCUAGUGGGCCAAAUCCUGG	24	54.2	38	1719	0.02
R18	GCCUAGUGGGCCAAAUCCUGG	21	61.9	383	1318	0.29
R19	UCCUGGAGGAAAUAAUUGA*GG* 1	21	42.9	0	1696	0
R20	CCUAGUGGGCCAAAUCCU	18	55.6	224	1336	0.17
R21	CCUAGUGGGCCAAAUCC	17	58.8	222	1110	0.2
R22	GUGGGCCAAAUCCUGGAGGAAAUA	24	50	415	722	0.57
R23	UAGUGGGCCAAAUCCUGGAG	20	55	116	1012	0.11
R24	GGCCAAAUCCUGGAGGAAAUAAUU	24	41.2	744	327	2.28
R25	AGUGGGCCAAAUCCUGGAGGA	21	57.1	740	286	2.59
R26	UAGUGGGCCAAAUCCUGGAGGAAA	24	50	815	208	3.92
R27	AAAUCCUGGAGGAAAUAAUUGA*GG* 1	24	37.5	0	967	0
R28	GGGCCAAAUCCUGGAGG	17	64.7	9	906	0.01
R29	UAGUGGGCCAAAUCCUGG	18	55.6	38	699	0.05
R30	AAAUCCUGGAGGAAAUAAUU	20	30	68	623	0.11
R31	CAAAUCCUGGAGGAAAUAAUU	21	33.3	564	117	4.82
R32	GGGCCAAAUCCUGGAGGAAAUAA	23	47.8	653	25	26.12
R33	GCCAAAUCCUGGAGG	15	60	26	647	0.04
R34	CCUAGUGGGCCAAAUCCUGG	20	60	435	237	1.84
R35	CUAGUGGGCCAAAUCCU	17	52.9	306	356	0.86
R36	GGCCAAAUCCUGGAGG	16	62.5	22	630	0.03
R37	AGUGGGCCAAAUCCUGG	17	58.8	25	551	0.05
R38	GUGGGCCAAAUCCUGG	16	62.5	510	62	8.23
R39	CUAGUGGGCCAAAUCCUGGA	20	55	322	240	1.34
R40	AAGCCUAGUGGGCCAAAUCCUG	22	54.5	485	44	11.02
R41	UAGUGGGCCAAAUCCU	16	50	43	484	0.09
R42	UGGGCCAAAUCCUGGAGGAA	20	55	491	32	15.34
R43	GGGCCAAAUCCUGGAGGAAAU	21	52.4	454	56	8.11
R44	AGCCUAGUGGGCCAAAUCCU	20	55	476	37	12.86
R45	AAUCCUGGAGGAAAUAAUU	19	31.6	39	466	0.08
R46	CUAGUGGGCCAAAUCCUGGAGG	22	59.1	38	457	0.08
R47	GCCAAAUCCUGGAGGAAAUA	20	45	301	166	1.81
R48	AAAAAGCCUAGUGGGCCAAAUCCU	24	45.8	429	10	42.9
R49	CCUAGUGGGCCAAAUCCUG	19	57.9	364	64	5.69
R50	UAGUGGGCCAAAUCCUG	17	52.9	120	307	0.39

1 Nucleotides in italic were derived from the loop sequence of *pART27-42*.

Among the six transgenic hairy lines with small RNA libraries sequenced, the four *pART27-42* lines had far more *16D10*-specific small RNAs than the two *pART27-271* lines (Table 1). *16D10*-specific reads for the two *pART27-271* lines were less than 100 while the four *pART27-42* lines had reads ranging from 40,000 to almost 70,000 (Table 1). Although all the four *pART27-42* lines showed better RKN resistance than the two *pART27-271* lines (Figure 2 and Table S1), such a large difference in the *16D10* small RNA production between *pART27-42* and *pART27-271* lines was unexpected. The low abundance of *16D10* small RNAs in the two *pART27-271* lines could be due to a low transcription level of the *16D10* dsRNAs and/or low efficiency in processing the long stem hairpin RNAs of *pART27-271* into siRNAs. Quantitative RT-PCRs were used to quantify the relative amount of the shared 42 bp fragment of the *16D10* gene in seven *pART27-42* and four *pART27-271* lines, including those that were used for the small RNA library construction. The relative transcription levels of 42 bp *16D10* in the *pART27-42* lines were 200–1500 times more of that in the *pART27-271* lines (Table S3). While we could not exclude other possibilities, low transcript level of dsRNAs was likely the main reason for the low abundance of *16D10*-specific siRNAs in the *pART27-271* hairy root lines.

### *16D10* dsRNA Stem was not Evenly Processed into siRNAs

**Figure 3:**
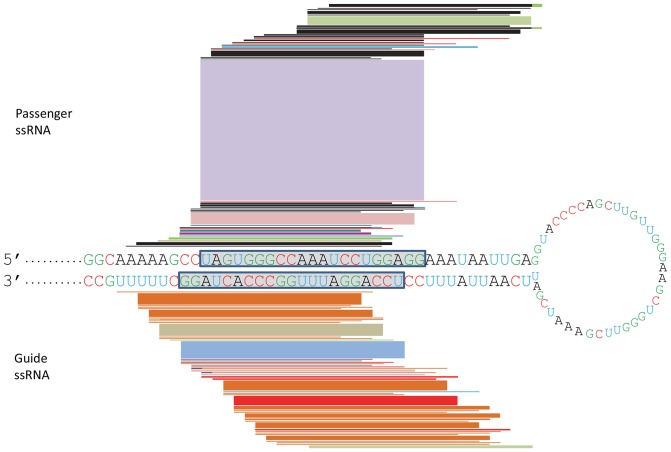
Distribution of the *16D10* small RNAs along the *pART27-42* hairpin stem. The hairpin structure of the *pART27-42* construct (the spliced-out intron not included) is presented with the *16D10* passenger ssRNAs aligned along the sense strand (above) and the guide ssRNAs aligned along the antisense strand (below). The relative small RNA abundance is graphically represented by the relative thickness of a block/line. Due to the limitation of graphic resolution, only those small RNAs with more than 200 reads were presented. The blocks with color variation in the 3′ ends indicate presence of mismatches. The “.” at the stem base represents the 5′ and 3′ overhangs due to the presence of cloning sites and other residual sequences from the *pART27-42* construct. The green arrows pointed to the first putative dicer cleavage site (21 nts away from the 5′ residue) and the red arrows pointed to the second putative dicer cleavage site (21 nts away from the first putative cleavage site). The grey boxes highlighted the siRNA duplex produced by these two cleavage events. Data for this figure were provided in Table 2 and Table S2.

**Figure 4:**
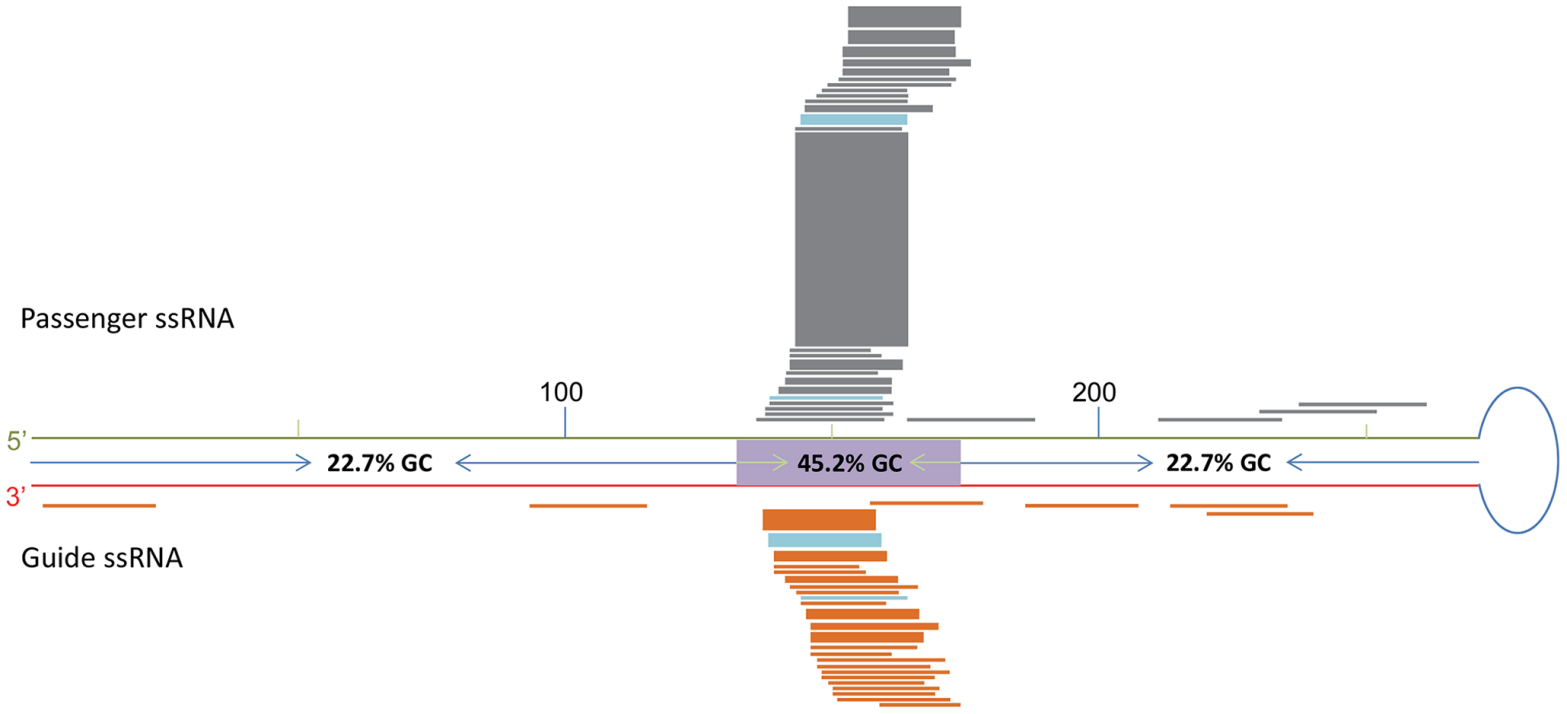
Distribution of the *16D10* small RNAs along the *pART27-271* hairpin stem. The schematic hairpin structure of the *pART27-271* construct is presented with the green line representing the sense strand, the red line representing the antisense strand, and the blue open circle representing the 39 nt loop. The numbers “100” and “200” along the stem indicate nucleotide positions from the 5′ stem end. The *16D10* 42 bp core coding region is marked as a purple box on the stem. GC content was marked for the stem base region, the middle core region, and loop terminus region. Each small RNA is represented as a block/line, with the thickness of a block/line indicating the relative abundance of a particular small RNA. Passenger and guide ssRNAs were aligned along the sense and antisense strands, respectively. Data for this figure were provided in Table S4.

Small RNAs from the four *pART27-42* lines were aligned to the 42 bp hairpin stem of the *pART27-42* construct to study the distribution pattern of *16D10* siRNAs (Figure 3). While the entire 42 bp stem was covered with small RNAs (Figure 3, Tables 2 and S2, and unpublished data), the top five most abundant small RNAs (R1-R5 in Table 2), representing 54% of the total *16D10* small RNAs, were located in a 27 bp region (Figure 3 and Table 2). The most abundant small RNA species, R1, had almost 80,000 reads comprising 38% of the total reads. In the *pART27-271* lines, more than 90% of the small RNAs were clustered around the same 42 bp core region as was observed in the *pART27-42* lines (Figure 4). It was not a surprise that several small RNAs which were abundant in the *pART27-271* lines were also found to be among the most abundant small RNA species in the *pART27-42* lines (Figures 3 and 4, Tables 2 and S4). For example, the small RNA species R1 was the most abundant small RNA in both *pART27-271* and *pART27-42* lines. Such similar distribution patterns of the *16D10*-specific small RNAs in both *pART27-42* and *pART27-271* suggested the presence of hot spots for siRNA generation in the *16D10* gene.

The stability of RNA secondary structure, largely determined by its GC content, might influence dsRNA processing and siRNA generation [40]. We compared the GC content of *16D10* small RNAs with that of the stem sequences of *pART27-42* and *pART27-271*. The top 50 small RNAs from *pART27-42* lines had an average GC content of 53.9%. In contrast, the GC content of the 42 bp stem sequence in *pART27-42* was 45.2% (Table 2). Similarly, small RNAs from *pART27-271* lines had an average GC content of 50.3%, whereas the GC content of the 271 bp stem sequence in *pART27-271* was much lower, only 26.2% (Figure 4). High GC content in the *16D10* small RNAs for both short and long dsRNA constructs suggested that GC content of dsRNAs might play an important role in transgene siRNA generation in plants.

Another interesting observation was that more siRNAs were produced from stem sequences near the loop terminus than the sequences near the stem base (Figures 3 and 4). For example, the small RNA species R6, R12 and R19 from *pART27-42* were aligned very close to the loop and each had more than one thousand reads (Table 2). In contrast, many small RNA species near the stem base had only a few or dozens of reads (Table 3). It was also interesting to note that R19 and R27 contained two nucleotides from the loop sequence (Table 2). This indicated that some loop sequences were involved in siRNA generation as well. For the 10 small RNAs that were unique to the *pART27-271* lines, eight were located in the 115 bp region proximate to the loop and only two were found in the 132 bp region toward the stem base (Figure 4). These observations suggested that, in addition to certain hot spots in the stem sequences for siRNA generation, the loop terminus sequences are more likely to be processed into siRNAs than the stem base sequences in the *16D10* dsRNA constructs.

**Table 3:** Putative *pART27-42*-derived siRNA duplexes, their 5′ end strength, and the reads of passenger and guide ssRNAs.

				5′ end strength of the siRNA duplex^1^ (ΔG, -kcal/mol)					
Duplex ID	Duplex sequence	small RNA IDas in Table 2		4 base pairings		2 base pairings		No. of ssRNA reads	
Sense	GGCAAAAAGCCUAGUGGGCCAAAUCCUGGAGGAAAUAAUUGA*GG* 2	Passenger		Sense		Sense		Passenger	
Antisense	CCGUUUUUCGGAUCACCCGGUUUAGGACCUCCUUUAUUAACU		Guide		Antisense		Antisense		Guide
**d1** 3	CAAAAAGCCUAGUGGGCCAAA	Rn^4^		4.9		3.1		8	
	CCGUUUUUCGGAUCACCCGGU		Rn		10.8		5.3		3
d2	AAAAAGCCUAGUGGGCCAAAU	Rn		4.3		1.9		2	
	CGUUUUUCGGAUCACCCGGUU		Rn		9.8		3.5		7
**d3**	AAAAGCCUAGUGGGCCAAAUC	Rn		4.5		1.9		20	
	GUUUUUCGGAUCACCCGGUUU		Rn		7.1		2.4		7
**d4**	AAAGCCUAGUGGGCCAAAUCC	R5		7		1.9		6	
	UUUUUCGGAUCACCCGGUUUA		Rn		4.6		1.8		59
d5	AAGCCUAGUGGGCCAAAUCCU	R9		9		2.7		70	
	UUUUCGGAUCACCCGGUUUAG		Rn		5.8		4		63
**d6**	AGCCUAGUGGGCCAAAUCCUG	R4		9.8		5.1		703	
	UUUCGGAUCACCCGGUUUAGG		R5		8.2		6.4		6414
**d7**	GCCUAGUGGGCCAAAUCCUGG	R18		9.2		6.4		1318	
	UUCGGAUCACCCGGUUUAGGA		R9		8.5		5.3		3981
d8	CCUAGUGGGCCAAAUCCUGGA	R2		7.8		5		800	
	UCGGAUCACCCGGUUUAGGAC		R4		10		4.8		6704
**d9**	CUAGUGGGCCAAAUCCUGGAG	R3		7.9		4.1		6340	
	CGGAUCACCCGGUUUAGGACC		R18		10.4		5.8		383
**d10**	UAGUGGGCCAAAUCCUGGAGG	R1		7.5		3.5		78930	
	GGAUCACCCGGUUUAGGACCU		R2		9.5		6		9745
d11	AGUGGGCCAAAUCCUGGAGGA	R25		9.2		4.5		286	
	GAUCACCCGGUUUAGGACCUC		R3		10		5.3		1393
d12	GUGGGCCAAAUCCUGGAGGAA	R7		10.9		5.1		90	
	AUCACCCGGUUUAGGACCUCC		R1		10.9		5.7		643
**d13**	UGGGCCAAAUCCUGGAGGAAA	R8		11.5		5.2		216	
	UCACCCGGUUUAGGACCUCCU		R25		10		6		740
**d14**	GGGCCAAAUCCUGGAGGAAAU	R43		13.8		7.5		56	
	CACCCGGUUUAGGACCUCCUU		R7		8.6		4		5603
**d15**	GGCCAAAUCCUGGAGGAAAUA	R10		11.8		7.1		118	
	ACCCGGUUUAGGACCUCCUUU		R8		7.6		2.4		5100
**d16**	GCCAAAUCCUGGAGGAAAUAA	R13		9.8		7.1		149	
	CCCGGUUUAGGACCUCCUUUA		R43		5.7		2.5		454
**d17**	CCAAAUCCUGGAGGAAAUAAU	Rn		6.9		5.1		138	
	CCGGUUUAGGACCUCCUUUAU		R10		4.6		2.8		3375
**d18**	CAAAUCCUGGAGGAAAUAAUU	R31		5.8		4		117	
	CGGUUUAGGACCUCCUUUAUU		R13		4.4		2.6		2231
d19	AAAUCCUGGAGGAAAUAAUUG	R12		5.7		2.5		2439	
	GGUUUAGGACCUCCUUUAUUA		R52		3.9		1.9		255
d20	AAUCCUGGAGGAAAUAAUUGA	R6		7.1		1.9		4657	
	GUUUAGGACCUCCUUUAUUAA		R31		4.5		2.5		564
**d21**	AUCCUGGAGGAAAUAAUUGA*G* 2	Rn		7.9		3.2		18	
	UUUAGGACCUCCUUUAUUAAC		R12		5.6		3.8		136
d22	UCCUGGAGGAAAUAAUUGA*GG* 2	R19		9.3		5.7		1696	
	UUAGGACCUCCUUUAUUAACU		R6		6.7		4.9		1649

1 The 5′ end strength (four or two base parings and one 3′ overhang) was calculated following the methods of Freier et al. [44] and Hutvagner [43].

2 The nucleotides in italic were derived from the loop sequence of *pART27-42*.

3 The siRNA duplexes, which followed the 5′ end strength rule (see text for detail) for producing guide and passenger ssRNAs, were in bold (4 bp calculation) or underlined (2 bp calculation).

4 “Rn” refers to small RNAs which were not among the top 50 small RNAs listed in Table 2.

### Relative Abundance of Guide and Passenger ssRNAs Varied with Small RNA Species and siRNA Duplexes

Once an siRNA duplex was produced from a dsRNA by a dicer-like enzyme, it needs to be unwound before the single strand RNA can be assembled into an RISC for its sequence-specific function. The unassembled complementary RNA strand will be degraded. An siRNA duplex has two 5′ ends. The ssRNA with less stable base pairing at the 5′ end will be more likely assembled into an RISC and thus be preserved and recovered by small RNA extraction [41]. The four base pairs at the 5′ end of an siRNA duplex are important determinants for the 5′ end thermodynamic strength while other studies suggested that the first two base pairs were most important [41], [42]. Many of the *16D10* small RNAs were recovered predominantly as either guide or passenger ssRNA (Figure 3, Tables 2 and S2). Among the top 100 small RNAs recovered from *pART27-42* lines, 65 of them had more than five times of guide or passenger ssRNAs over the complementary ssRNAs (guide/passenger ssRNA ratio bigger than 5 or smaller than 0.2). To determine whether or not the relative abundance of guide and passenger ssRNAs from an siRNA duplex is related to its 5′ end thermodynamic strength, we analyzed the 21-nt small RNA species, which were the most dominant type of *16D10* small RNAs, from the *pART27-42* hairy root lines. The putative 21-nt siRNA duplexes were aligned to the 42**bp stem and numbered in an ascending order from d1 (duplex 1) to d22 (duplex 22) starting from the stem base (Table 3). The 5′ end strength for two or four base pairings plus one 3′ overhang was calculated for these 21-nt siRNA duplexes using the nearest-neighbor method as previously reported [43], [44]. The relative stability of the 5′ ends of an siRNA duplex could largely explain the relative abundance of the guide and passenger ssRNAs originated from the same siRNA duplex. Among the 22 putative siRNA duplexes, 17 had predicted relative abundance of guide and passenger ssRNAs on the basis of their 5′ end strength calculated from either two or four base pairings (Table 3). For example, the putative duplex 10 in Table 3 contains the two most abundant 21-nt small RNA species (R1 and R2 in Table 2). The 5′ end strength for the sense strand “UAGU” (-7.5 kcal/mol) is weaker than that of the antisense strand “UCCA” (-9.5 kcal/mol). Indeed, the count of the passenger ssRNA (R1 passenger, 78,930) was about eight times that for the guide ssRNA (R2 guide, 9,745). Similarly, the siRNA duplex 9, which shifted one nucleotide towards the stem base from duplex 10, also produced much more passenger ssRNA (6,340) than the guide ssRNA (383) due to their difference in the 5′ end strength (“-7.9” via “-10.4”). Interestingly, the total reads from duplex 10 were ten times more than that from duplex 9 while these two siRNA duplexes differed in only one nucleotide/position, suggesting that the abundance or stability of an siRNA may also depend on its location, composition, and/or internal thermodynamic property, in addition to the 5′ end strength. Indeed, the relative abundance of guide and passenger ssRNAs in five out of the 22 siRNA duplexes can’t be simply explained by the theory of thermodynamic strength of the 5′ ends (Table 3). For example, the siRNA duplex 12 produced guide ssRNAs seven times more of the passenger ssRNAs while their 5′ ends had similar thermodynamic strength (Table 3). Duplex 11 did not follow this 5′ end strength rule either, producing guide ssRNAs almost five times of the passenger ssRNAs, in spite of that the guide strand had a stronger 5′ end strength (“-10.0” via “-9.2”). These exceptions suggest that additional mechanisms might be responsible for determining the relative abundance between guide and passenger ssRNAs. It is also possible that different ssRNAs might be recovered differently during RNA extraction and small RNA library preparation [45].

## Discussion

Hairy root systems have been used for testing nematode parasitism in soybean [15], tomato [10], cotton [46], and plum [39]. Hairy roots have also been used in functional genomics research, as they offer a quick means for testing gene functions without going through the lengthy process of producing stable transgenic plants. Such a hairy root system is especially useful for crops like grapes, as generation of a stable transgenic grapevine takes at least 18 months or even longer. We developed a system for producing transgenic grape hairy roots for functional genomics research [34] and the system was used in the current study. While grape hairy roots offered a unique system for evaluation of the efficacy of *16D10* dsRNAs in suppressing RKNs in this study, there were some challenges in maximizing the utility of the system. One significant challenge was the wide range of variation in root morphology and proliferation ability among different hairy root lines. Such variation inevitably impacted the quality and vigor of the root lines, presenting difficulties for maintaining and obtaining enough uniform roots for evaluation in multiple experiments and replicates. Furthermore, we observed that thick hairy roots appeared more resistant to RKN infection. *pART27-271* line 20 was one such line as discussed in the results. Similar observations were noted in some other lines as well (unpublished data). These challenges emphasize the importance of selecting hairy roots with uniform size and vigor for an experiment. Significant variation in hairy root morphology was also reported in cotton [46]. The cause is unknown for producing hairy roots with a wide range of root morphology and growth vigor. One possible explanation is that the levels of auxin produced by the *rol* gene from the hairy-root-inducing plasmid of *A. rhizogenes* could be different among different hairy root lines, resulting in hairy roots with different vigor and morphology.

We evaluated two stem configurations, one with a 42 bp stem and the other with a 271 bp stem. Our small RNA sequencing data revealed that transgenic hairy roots carrying the short 42 bp stem produced much more *16D10*-specific siRNAs than those carrying the long 271 bp stem. This result was in agreement with what was observed in mammalian cells: a short stem is more effective than a long stem for high-efficient RNAi delivery [20]. While the exact molecular mechanism for more efficient siRNA production by the short hairpin construct than the long hairpin construct in this study was not elucidated, our quantitative RT-PCR results suggested that the short 42 bp stem was far more abundantly transcribed and/or accumulated than the long one (Table S3). This result is in agreement with the findings in mammalian cells that the shRNAs of longer stems had reduced siRNA activities and also reduced dsRNA expression/accumulation based on northern blot analysis [21].

Several siRNA hot spots were revealed regarding the distribution patterns of *16D10* siRNAs along the hairpin stems. These hot spots had higher GC content than the rest of the stem sequences. Hot spots for siRNA production and high GC content of siRNAs were also observed in other plant RNAi research. For example, hot spots for siRNA production were identified along the 400 bp stem sequence of a dsRNA *GFP* construct evaluated in transgenic *Arabidopsis* [47]. In a different study, 40 *GFP*-derived small RNAs were cloned from tobacco leaves transiently transformed with *35S::dsGFP* construct [48] and these cloned *GFP* small RNAs showed a higher GC content than the *GFP* stem sequence (48.2% via 43.5%). Similarly, in a study of plant virus-derived siRNAs, it was found that the virus-derived siRNAs from infected plants had a higher GC content than the virus genome (52.6%–53.6% for siRNAs via 45.7% for the virus genome) [40], [49]. These observations were consistent with the fact that most small RNAs from the *pART27-271* construct were mapped to the 42 bp GC-rich core region (Figure 4) and further support the previous observation that plant dicer-like enzymes prefer GC rich sequences [40], [49].

In addition to GC rich hot spots for *16D10* siRNA production, it appeared that the loop terminus sequences in both *pART27-42* and *pART27-271* were more likely to be processed into siRNAs than the base terminus sequences. This observation, however, is not consistent with the findings in mammalian RNAi research. Several RNAi studies in mammalian cells suggested that dsRNA processing started from stem base, thus sequences at the stem base were more likely to be processed into siRNAs [20], [21], [50], [51]. This inconsistence could be due to several reasons. One of the possible reasons is that the conclusions from these mammalian RNAi studies were based on northern blot results with probes not covering the whole stem sequences. Therefore, not all the small RNA species produced from shRNAs were examined. In our study, we were able to examine most of the small RNAs, if not all, using the high throughput sequencing technique. The second possible reason is that our dsRNA construct configurations were quite different from that examined in mammalian cells. For example, the loop size in our dsRNA constructs was much bigger (39 nts) (Figures 3 and 4) than that in most shRNAs evaluated in the mammalian studies (1–15 nts). It has been known that both loop length and sequence can affect shRNA silencing efficacy [18], [52], [53]. In addition to difference in loop size and sequence, the 5′ and 3′ overhangs were also different. Most evaluated mammalian shRNAs had 3′ overhangs of two nucleotides and no 5′ overhang. Such shRNA configurations are preferred substrates for dicer enzymes. Longer 5′ or 3′ overhangs at the stem base often reduced the efficiency of dsRNA processing and silencing [54]. The 5′ and 3′ overhangs could also have impact on the dicer cleavage specificity and efficiency since both 5′ and 3′ residues function as anchors for dicers [55]. The dsRNA constructs in our study contained about 10 nt overhangs for the 5′ end and more than 10 nt overhangs for the 3′ ends due to the presence of cloning sites, some poly A tail and/or other residual sequences. These long overhangs may contribute to the different small RNA distribution patterns between our and the mammalian studies. If plant dicer-like enzymes process the *16D10* dsRNA from stem base, the first cleavage product of the dsRNA stem sequences (21 nts from the 5′ residue) would not be stable due to the long 5′ and 3′ overhangs. In addition, these unstable cleavage products might not be able to load efficiently into RISCs and thus less small RNAs would be preserved from the stem base sequences. We did recover some small RNAs located at the junctions of the 42 bp stem with the 5′ overhang, the 3′ overhang, or the loop sequence when the whole hairpin sequence, including the 42 bp stem, the overhangs and the loop sequence, was used as the reference to map the small RNAs located at these junctions (R19 and R27 in Table 2, and unpublished data).

Plant small RNAs were often classified as sense or antisense RNAs on the basis of their orientations on the dsRNA stems of RNAi constructs or the original genomes of interest [40], [47], [49], [56]. Little is known about why certain small RNAs were predominantly produced as sense or antisense RNAs. In this study, we examined the putative siRNA duplexes and their corresponding sense and antisense ssRNAs in the *pART27-42* lines. The *16D10* small RNA profiles in this study largely support the previous observations that ssRNAs with less stable 5′ ends were more likely to be retained, compared to their complementary ssRNAs from the same siRNA duplexes [41], [42]. Another interesting observation was that the most abundant *16D10*-specific small RNA was the passenger ssRNA, R1, which counted for more than 38% of the total *16D10*-specific small RNAs. This passenger ssRNA obviously didn’t contribute to the *16D10* gene silencing. Why did this particular ssRNA occur at such a high frequency? The location of this ssRNA on the dsRNA stem may offer a good explanation. As discussed earlier, the first putative plant dicer cleavage site would be 21 nts away from the 5′ residue in the *pART27-42* hairpin RNAs (Figure 3), if plant dicer-like enzymes process dsRNA from stem base using 5′ counting rule as human dicer enzymes do [55]. The first cleavage would produce unstable duplexes due to the long overhangs and also leave an optimal 2 nt 3′ overhangs for the next dicer cleavage. Then the next most likely cleavage site would be 21 nts away from the first cleavage site. This second cleavage would produce the putative siRNA duplex 10 from which the R1 ssRNAs were generated (Figure 3). Our small RNA data revealed that siRNA duplex 10 was indeed the most abundant siRNA duplex produced from the *pART27-42* construct, suggesting that plant dicer-like enzymes use the 5′ counting rule as the human dicer enzymes do. This would also explain why small RNAs from the duplex 9 and duplex 11, which were just one nucleotide away from duplex 10, were much less abundant (Table 3). Another important factor for the R1 abundance, as explained earlier, is that this sense ssRNA (R1passenger) had a less stable 5′ end than its counterpart antisense ssRNA (R2 guide) and thus was more likely to be preserved in RISC (Table 3). Additional explanation might be that this particular small RNA “UAGUGGGCCAAAUCCUGGAGG” had low internal energy due to the presence of “AAAU” in the middle of its sequence, since low internal energy was another thermodynamic signature for most miRNAs and functional siRNAs [42]. It would be interesting to investigate whether or not the ratio of passenger/guide ssRNAs could be altered by modifying the sense sequence composition and the 5′ overhangs in the *pART27-42* dsRNA construct.

The *16D10*-specific small RNAs produced by *pART27-271* lines were less than 0.2% of that by the *pART27-42* lines. However, the *pART27-271* lines still exhibited a good level of RKN resistance. One possible explanation is that *pART27-271 16D10* small RNAs might be recovered at low efficiency during small RNA extraction due to their low GC content beyond the 42 bp core region (9-33%) (Figure 4 and Table S4). Potential negative influence of low GC content on small RNA extraction was previously reported [45]. An alternative explanation is that some *pART27-271 16D10* small RNAs were highly potent in producing silencing effect. These small RNAs with low GC content might have better access to the target gene and thus offer better target suppression as suggested by others [27], [28], [30], [57], [58]. In some animal RNAi studies, it was also found that the most potent siRNAs do not need to be expressed at high levels [30], [31]. Recently, an interesting study showed that transgenic cucumber plants, in which the transformed hairpin construct targeting a virus gene contained hot spots for producing anti-viral siRNAs, were not only immune to the wild-type virus of interest but also resistant to mutant viruses whose gene sequences corresponding to the hot spots were mutated [56]. The study strongly suggested that majority of the transgene siRNAs in the cucumber plants might not be functional for target gene suppression or not many siRNAs were required for effective gene silencing. This raised an interesting and important question of how adequately to determine silencing efficacy of individual siRNAs. Perhaps the reporter-sensor screening system routinely performed in mammalian RNAi studies for selection of the most potent siRNAs [27], [30], [59] can offer some help in this regard.

This study demonstrated the efficacy of *16D10* siRNAs in inhibiting RKN infection in transgenic grape hairy roots and provided first proof-of-concept for developing transgenic grapevines for resistance to RKNs via an RNAi approach. It also provided some insights into optimizing various components of RNAi constructs for enhancing RNAi efficiency. Using a short stem hairpin structure and examining thermodynamic properties of the stem sequence are among the important factors to consider for increasing the chance of producing abundant and effective guide ssRNAs.

## Supporting Information

Table S1 **Mean fresh hairy root weight and numbers of nematode eggs per hairy root and per gram root observed in transgenic grape hairy root lines infected with root-knot nematodes.**
(XLSX)

Table S2 **The top 51–100 small RNAs from four** ***pART27-42*** **transgenic hairy root lines.**
(XLSX)

Table S3 **Relative expression levels of the** ***16D10*** **gene, determined by Q-RT-PCR, in four** ***pART27-271*** **and seven** ***pART27-42*** **transgenic hairy root lines.**
(XLSX)

Table S4 **The small RNAs recovered from** ***pART27-27*** **1hairy root lines.**
(XLSX)
